# Urine Testing in a Local Drug and Alcohol Service: How Has the COVID-19 Pandemic Affected the Frequency of Urine Testing in Patients With Opiate Addiction?

**DOI:** 10.1192/bjo.2022.276

**Published:** 2022-06-20

**Authors:** John Barker, Olawale Lagundoye, Thomas Nield

**Affiliations:** 1 The University of Sheffield, Sheffield, United Kingdom; 2 Sheffield Health and Social Care NHS Foundation Trust, Sheffield, United Kingdom

## Abstract

**Aims:**

When a dependant opiate user seeks help from a substance misuse service, it is vital that some form of drug testing is conducted. This is commonly a urine test and will show the patient's drug use over recent days and is used as a basis to guide treatment. All patients should have a urine sample collected and tested on the same day as their initial appointment, however, we hypothesised that the switch to remote consultations would have reduced the number of urine tests conducted post-pandemic.

**Methods:**

All the patients initially assessed by the substance misuse services for treatment of opiate addiction within the Sheffield Health and Social Care NHS Foundation Trust between 01/03/19 (1 year prior to the pandemic) and 01/03/21 (one year after the pandemic).

The resultant sample contained 1403 patients: 739 patients were referred to Sheffield substance misuse services prior to the start of the COVID-19 pandemic; 664 patients were referred to Sheffield substance misuse services during or after the start of the COVID-19 pandemic.

An algorithm was developed to allow interrogation of the electronic notes to record whether or not urine samples were taken and recorded in the relevant section of the patient's electronic record. This information was then transferred to an Excel spreadsheet.

**Results:**

The proportion of patients who had a urine test on the same day as their initial appointment was significantly higher in the year prior to the pandemic (79.0%) than the subsequent year (35.8%).

**Conclusion:**

36% of the sample in the year subsequent to the pandemic had a urine test the day after their initial assessment, rather than on the same day. This delay in urine analysis can be attributed to the large number of initial appointments being conducted via telephone during the COVID-19 pandemic. This led to a delay in getting patients into clinic to give a urine sample. However, the remaining 64% of patients had no sample recorded in their notes in the appropriate proforma. Suggestions for improvement are to include a session on urinalysis as part of the weekly CPD to drive an improvement in this score back to pre-pandemic levels.

